# Transactions of the Twenty-sixth Session of the Homœopathic Medical Society of Penna.

**Published:** 1891-11

**Authors:** 


### BOOK NOTICES.

TRANSACTIONS OF THE TWENTY-SIXTH SESSION OF THE HOMŒOPATHIC MEDICAL SOCIETY OF THE STATE OF PENNSYLVANIA. Held at Philadelphia, September 17th–19th,

1891. Philadelphia: Sherman & Co., Printers, Seventh and Cherry Streets, 1891.

This little volume makes a very creditable appearance. The different addresses by the officers are good and to the point. And there is a determined effort on the part of the different Bureaus to give only that which is homœopathic. "Well done, ye good and faithful servants." W. S.

